# A Systematic Review of Attentional Focus Strategies in Weightlifting

**DOI:** 10.3389/fspor.2019.00007

**Published:** 2019-08-09

**Authors:** David L. Neumann

**Affiliations:** School of Applied Psychology, Griffith University, Gold Coast, QLD, Australia

**Keywords:** attention, weightlifting, concentration, performance, strength training, attentional focus

## Abstract

The way an athlete focuses their attention when lifting a weight has the potential to influence strength development during training and performance outcomes during competition. The effects of attentional focus strategies during weightlifting tasks was investigated through a systematic review. Major databases (SportDISCUS, PsycINFO, Scopus) were searched using key terms relevant to attentional focus and weightlifting and reference lists of identified articles were also searched. Following screening, 16 articles were retained for analysis. The review showed that researchers have recruited experienced and novice weightlifters of both genders in their studies, although male experienced weightlifters are the most commonly studied demographic. Weightlifting tasks have varied from bench press, biceps curls, squats, and leg extensions with some studies using measures of force production against a force plate. The predominant manipulations have been between internal-associative and external-associative foci. An external attentional focus has shown to be beneficial in terms of movement economy as reflected in a variety of outcome measures. The results are interpreted within the framework provided by the Constrained Action Hypothesis and more generally the advantages of an external attentional focus for motor skill learning. An external focus of attention promotes automatic control of actions, thus preventing the motor system being constrained by conscious cognitive control. Implications for informing training programs for athletes and for advising athletes to maximize performance during competition are discussed.

## Introduction

The action of lifting a weighted apparatus is ubiquitus in sport and exercise. It is used during training to develop muscle strength, muscle mass, and joint strength. It is is also a competitive sport in its own right, as reflected in its Olympic Games status and the formation of national and international governing bodies. The sport of weightlifting requires lifts of the snatch and the clean and jerk with athletes aiming to lift the heavest weight for their division during competition. Other competitive events, often referred to as powerlifting, require the deadlift, squat, and bench press. Weightlifting is also a key component of training for other sports and as part of a physical exercise program. In these situations, there are a multitude of different types of lifts according to the muscles required, equipment used, and the speed, duration, and complexity of the movements.

The physical nature of weightlifting has naturally led to research on physical factors, such as physiology, biomechanics, diet, and injury. Comparatively less work has been conducted on the psychological processes associated with weightlifting. The psychology of weightlifting has been examined from various perspectives, including self-efficacy, intention, and self-regulation behaviors (Rhodes et al., 2017), mindfulness and contemplative movement (Vernon, 2018), and instruction techniques (Milanese et al., 2017). Attentional focus is another psychological factor that has potentially important implications for learning and performance in weightlifting.

Attentional focus, in the context of sport and exercise performance, refers to the process in which the athlete allocates mental resources to cues, stimuli, or states. Attentional focus is commonly classified along one or more dimensions. Nideffer (1976) proposed two dimensions of direction (internal or external) and width (broad or narrow). Stevinson and Biddle (1998) also proposed two dimensions, although they divided attentional foci along task-relevance (association or dissociation) and direction (internal or external). Two dimensional schemes such as these will allow for a particular attentional focus to reflect a combination of the two dimensions. For instance, the task-relevance and direction scheme results in four combinations of internal association (e.g., muscle fatigue, breathing, pain), internal dissociation (e.g., daydreams, mental puzzles, recalling memories), external association (e.g, split times, distance markers, targets), and external dissociation (e.g., scenery, crowd, listening to music).

Subsequent classification schemes have extended upon the task-relevance (or association) and direction dimensions of Stevinson and Biddle (1998). Wininger and Gieske (2010), for example, divided a task-relevance internal foci into bodily sensations, task-relevant thoughts, and self-talk. Brick et al. (2014) used two internal association categories of internal sensory monitoring and active self-regulation. The external association combination has also been conceptualized in different ways, such as a focus on the movement effect (Wulf, 2013). The dimensions or specific categories in a dimension may be more relevant for some types of sports than others. For example, the scheme proposed by Brick et al. (2014) provides an excellent framework for endurance sports like running, cycling, and rowing.

Attentional focus in weightlifting has been largely investigated from the attentional focus strategies of internal association and external association (usually simply referred to as internal and external foci). This approach stems from the influential work by Wulf et al. demonstrating the performance benefits of an external attentional focus over an internal attentional focus in ski-simulator and balancing tasks (Wulf et al., 1998). The external attentional focus benefits were subsequently extended to other motor and sport-related tasks (for reviews, see Wulf, 2007, 2013; Marchant, 2011; Lohse et al., 2012; Wulf and Lewthwaite, 2016). The research has generally shown that focussing away from the body and on the intended movement effect (external focus) produces superior learning and performance outcomes than focussing toward the body (internal focus). Moreover, this effect seems to be due to a relative improvement in performance with an external focus, rather than a relative impairment of performance with an internal focus, because an external focus will typically produce better outcomes than no specific attentional focus instructions.

The benefits of an external focus of attention for motor learning and performance has been reflected in a range of tasks and outcome measures (for details see Wulf and Lewthwaite, 2016). Benefits have been observed in movement effectiveness (e.g., better balance, higher accuracy), movement efficiency (e.g., reduced muscular activity, higher peak force, greater speed, longer endurance), better movement form, and more automatic and fluid movements. The potential for an external focus of attention to enhance movement efficiency is of particular relevance for weightlifting. For example, it may allow an athlete to lift the same weight through less muscular effort. Conversely, and more importantly for competition, it may allow an athlete to lift a heavier weight than would otherwise be possible when no specific attentional focus is adopted.

The present review examined research on attentional focus strategies during weightlifting. A systematic literature review was conducted in which relevant electronic databases were searched using key terms. Search results were screened to yield a final set of articles for coding and analysis. The review aimed to answer the following questions:

1. What types of weightlifting tasks and equipment are used in research?
2. What are the characteristics of the participants who have been studied?
3. What have been the aims, methods, conditions, measures and key findings of research?
4. What theoretical framework has been used to guide the research and interpret the findings?

Following a review of these questions, it was hoped to develop some general principles from what is known about attentional focus in weightlifting. Practical applications and suggestions for research are also offered.

## Methods

The Preferred Reporting Items for Systematic Review and Meta-Analyses (PRISMA) guidelines were followed for the literature search (Liberati et al., 2009), and the rules of inclusion and exclusion described by Meline (2006) were applied. Initially, the SPORTDiscus, PsycINFO, and Scopus databases were searched. The terms used in conducting the search included: (“focus of attention” OR “attentional focus” OR “attentional focusing”) AND (“weight lifting” OR weightlifting OR “weight training” OR “strength training” OR “force production” OR “motor control”) AND (internal OR external OR association OR dissociation OR associative OR dissociative). The search was not limited by date of publication and included all articles available at time of search (October, 2018). Additionally, to identify articles that may have been missed due to inconsistent use of terms (e.g., “attentional focus” vs. “focus of attention”), the reference lists of all articles initially selected for inclusion from the database search were examined.

**Figure 1:**
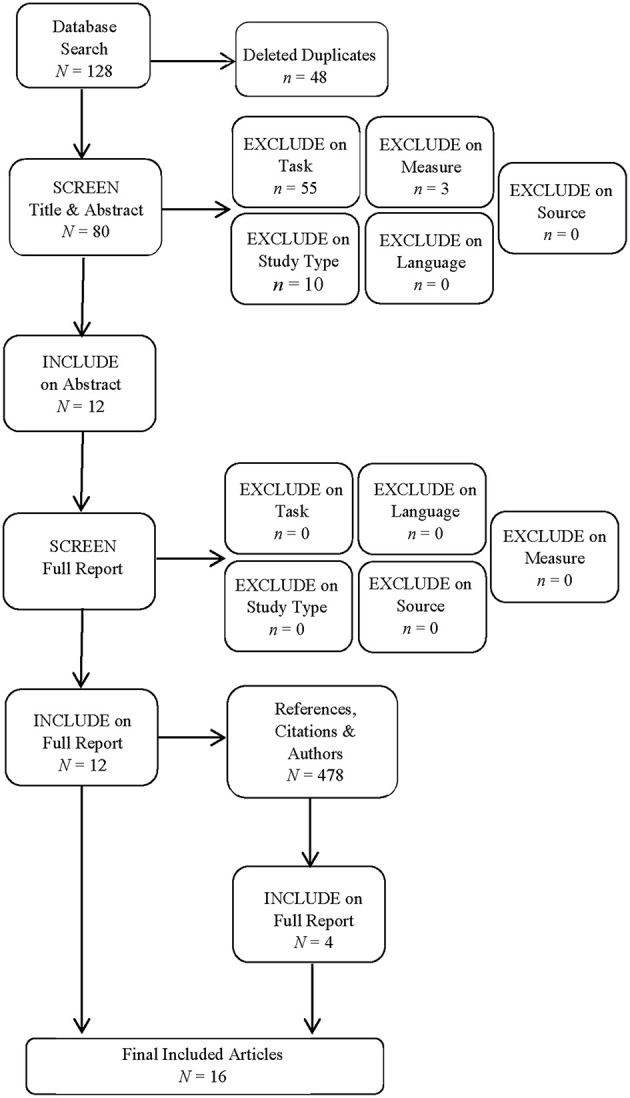
Search results and outcomes of the screening process.

The results from the literature search and screening are shown in Figure 1. The database search resulted in 27 articles from PsychINFO database, 46 articles from SPORTDiscus database, and 55 articles from Scopus database, totaling 128 articles. Following the removal of duplicates, this number was reduced to 80 unique articles. Articles were then screened for exclusion or inclusion in a two-step process: title and abstract (step 1) and the full article (step 2). Articles were excluded based on the following criteria: language (not published in English language), source (a dissertation, thesis, abstract only, magazine article, or from a non-peer reviewed source), study type (review, meta-analysis, commentary, letters, or any non-empirical article), did not measure or manipulate attentional foci, did not examine movement against a weight or force plate, or did not include a measure of physical performance or physiological activity.

Following this selection process, a total of 12 articles from the database search, with a further four articles identified following the examiniation of the selected articles reference lists were obtained. As such, a total of 16 articles were included in the systematic review. Table 1 shows the citation metrics for the articles that were published in journals present in the Scimago Journal & Country Rank database. The mean 2-year impact factor was 1.90 (range 0.968–2.717, *SD* = 0.54), the mean journal h-index was 86.33 (range 42–117, *SD* = 22.64), and seven journals were Q1 ranked. These citation metrics suggest that the journals that published this research were of moderate to high quality. The articles were coded by study characteristics (aims, conditions/groups, outcome measures, and key findings), participant characteristics (sample size, age, gender, weight lifting experience, and location), and task characteristics (exercise/movement completed and equipment used).

**Table 1:** Publication details of the studies selected for review.

**References**	**Journal name**	**2-year impact factor**	**Journal h-index**	**Quartile**
Calatayud et al., 2018a	Journal of sports sciences	2.715	117	Q1
Calatayud et al., 2018b	Perceptual and motor skills	0.989	60	Q4
Greig and Marchant, 2014	Human movement science	1.956	80	Q2
Halperin et al., 2016	Journal of strength and conditioning research	2.340	108	Q1
Kristiansen et al., 2018	Journal of strength and conditioning research	2.340	108	Q1
Lohse, 2012	Human movement science	1.956	80	Q2
Lohse et al., 2011	Journal of motor behavior	1.328	63	Q3
Lohse and Sherwood, 2012	Acta psychologica	1.632	88	Q1
Marchant et al., 2008	Athletic insight	–	–	–
Marchant et al., 2009	Journal of strength and conditioning research	2.340	108	Q1
Marchant et al., 2011	Research quarterly for exercise and sport	2.01	82	Q2
Marchant and Greig, 2017	Human movement science	1.956	80	Q2
Neumann and Heng, 2011	Journal of psychophysiology	0.968	42	Q3
Schutts et al., 2017	Journal of strength and conditioning research	2.340	108	Q1
Snyder and Fry, 2012	Journal of strength and conditioning research	2.340	108	Q1
Vance et al., 2004	Journal of motor behavior	1.328	63	Q3
	Mean (SD)	1.90 (0.54)	86.33 (22.64)	

*Citation metrics of 2-year impact factor, journal h-index, and quartile ranking were obtained from Scimago Journal and Country Rank database*.

## Results

### Tasks and Equipment Used in Research

The types of weightlifting tasks and equipment used in research on attentional focus strategies are shown in Table 2. As can be seen, the bench press (Marchant et al., 2011; Snyder and Fry, 2012; Calatayud et al., 2018a,b; Kristiansen et al., 2018), force plate (Lohse et al., 2011; Lohse, 2012; Lohse and Sherwood, 2012) and bicep curls (Vance et al., 2004; Marchant et al., 2008, 2009; Neumann and Heng, 2011) have been the most commonly used. The bench press and bicep curls are advantageous because they involve relatively simple movements and effectively isolate key muscles. The bench press is also a powerlifting event, and so enhances the real-world relevance of outcomes for competition. The snatch was used by Schutts et al. (2017), which is the only study found in the search to have used an Olympic weightlifting event.

**Table 2:** Characteristics of the task and equipment used.

**References**	**Task**	**Equipment**
Calatayud et al., 2018a	Bench press	Barbell
Calatayud et al., 2018b	Bench press	Barbell
Greig and Marchant, 2014	Bicep curl	Isokinetic dynamometer (Biodex, System 3)
Halperin et al., 2016	Isometric midthigh pull	Barbell and 9290AD quattro jump force plate
Kristiansen et al., 2018	Bench press	Barbell
Lohse, 2012	Force production—foot pressing	Force plate
Lohse et al., 2011	Force Production—Foot Pressing	Force plate
Lohse and Sherwood, 2012		
Experiment 1	Force production—foot pressing	Force plate
Experiment 2	Force production—foot pressing	Force plate
Marchant et al., 2008	Bicep curls	Isokinetic dynamometer (biodex, system 3)
Marchant et al., 2009	Bicep curls	Isokinetic dynamometer (biodex, system 3)
Marchant et al., 2011		
Exercise 1	Assisted bench press: 40 kg for men, 20 kg for women	Smith machine
Exercise 2	Bench press: 75% of 1-RM	Standard bench and barbell
Exercise 3	Back Squat in high bar position: 75% of 1-RM	Standard barbell
Marchant and Greig, 2017	Leg extension	Isokinetic dynamometer (biodex, system 3)
Neumann and Heng, 2011	Bicep curls	Dumbbell set with changeable disc weights
Schutts et al., 2017	Snatch: 80% of 1-RM	Standard barbell
Snyder and Fry, 2012	Bench press: 50 and 80% of 1-RM	Standard barbell and bench
Vance et al., 2004	Bicep curls-−50% of 1-RM	Weighted barbell

Lohse et al. (2011), Lohse (2012), and Lohse and Sherwood (2012) examined force production when participants pushed with their feet against a force plate. The apparatus allows for the investigation of motor planning (Lohse, 2012), as well as intermuscular coordination (e.g., co-contraction of muscles) and intramuscular coordination (e.g., motor-unit recruitment) under different types of attentional foci (Lohse et al., 2011). The use of a force plate is thus a useful complement to free weights in research. Although not included following the screening process, research has also examined attentional focus effects on muscle activity during a sit up task (Neumann and Brown, 2013), which was a task that did not involve muscular force against any apparatus. There are thus a wide variety of tasks that researchers have used to examine attentional foci at the neuromuscular level.

### Participant Characteristics

The characteristics of the participants who have been studied in research are shown in Table 3. Most studies have used both male and female participants, with some studies restricting their sample to males only. No studies exclusively used female participants. Although sex does not typically moderate attentional focus effects (but for examples see Becker and Smith, 2013; Flôres et al., 2016; Emad et al., 2017), it is still an important empirical question on whether sex differences exist for weightlifting. It is thus recommended that future research include both sexes in research when possible to ensure generalisability of findings and that analyses are conducted to check for sex differences.

**Table 3:** Sample size and participant characteristics.

**References**	***N***	**Gender**	**Age in years (*SD*)**	**Experience**	**Location**
Calatayud et al., 2018a	18	Male	*M* = 31 (8)	Experienced	Denmark
Calatayud et al., 2018b	18	Male	*M* = 31 (8)	Experienced	Denmark
Greig and Marchant, 2014	25	Both	*M* = 23.53 (1.76)	Inexperienced	England
Halperin et al., 2016	22	Both	*M* = 22.5 (3.3) Range: 17–28	Experienced	Australia
Kristiansen et al., 2018	21	Male	*M* = 24.5 (2.2)	Experienced	Denmark
Lohse, 2012	24	Both	Undergraduates	Unknown	United States
Lohse et al., 2011	12	Both	Undergraduates	Unknown	United States
Lohse and Sherwood, 2012					
Experiment 1	12	Both	Undergraduates	Unknown	United States
Experiment 2	12	Both	Undergraduates	Unknown	United States
Marchant et al., 2008	29	Both	*M* = 19.6 (1.3)	Experienced	England
Marchant et al., 2009	25	Both	*M* = 22.72	Inexperienced	England
Marchant et al., 2011					
Assisted bench press	23	Both	*M* = 30.87 (12.27)	Experienced	England
Bench press	17	Male	*M* = 20.82 (1.42)	Experienced	England
Back squat	17	Male	*M* = 20.82 (1.42)	Experienced	England
Marchant and Greig, 2017	20	Both	*M* = 20.2 (1.47)	Experienced	England
Neumann and Heng, 2011					
Group 1	16	Both	Male: *M* = 23.14 (4.28) Female: *M* = 29 (11.31)	Novice	Australia
Group 2	14	Both	Male: *M* = 24.31 (3.30) Female: 22	Experienced	Australia
Schutts et al., 2017	12	Both	*M* = 23.7 (2.9)	Experienced	United States
Snyder and Fry, 2012	11	Male	Undergraduates	Experienced	United States
Vance et al., 2004					
Experiment 1	11	Male	*M* = 26.00 (6.00)	Experienced	United States
Experiment 2	12	Both	Not provided	Experienced	United States

Participants have tended to be experienced in weightlifting, although a sizeable portion of studies did not specify the participant experience level. Experienced participants are more likely to be well-practiced and to have develop automaticity in movements. Given the notion that an external focus of attention facilitates automatic motor processes (Wulf and Lewthwaite, 2016) it may be expected that experienced participants are particularly likely to benefit from an external focus of attention than an internal one. Novice participants have also shown learning and performance benefits from an external attentional focus across a range of tasks (Wulf and Lewthwaite, 2016). However, there have been exceptions (e.g., Perkins-Ceccato et al., 2003). In line with the recommendation of Greig and Marchant (2014), further research is needed that tests for differences between experienced and novice participants in attentional focus effects.

Other characteristics of the participants have shown that many studies have recruited undergraduate students or participants aged 20–25 years on average. While some studies have used older samples, these have had a mean age no older than 31 years. Future research could recruit older adult samples to ensure the generality of the findings across a wide age range. Similarly, no studies have recruited younger participants, such as adolescents and children, and it remains to be determined whether study findings can be replicated with a young age group. Most studies have been conducted in Western countries, most noteably the USA, England, and Denmark. Finally, the sample sizes used in research has been relatively small. Samples have varied from 11 to 29 participants with a mean of 17.67 participants. It is recommended that researchers recruit larger samples to ensure that there is sufficient statistical power, to minimize the reporting of spurious findings, and to ensure generality of findings. In summary, reseach has used relatively small samples with participants typically comprised of young males from Western countries who are experienced in the sport of weightlifting.

### Aims, Methods, and Key Findings

A summary of the aims, methods, conditions, measures, and main findings in the experiments reported in the 16 studies reviewed is provided in Table 4. An external focus of attention has produced lower EMG activity (peak EMG, average EMG, or integrated EMG) than an internal focus of attention in several studies (Vance et al., 2004; Marchant et al., 2008, 2009; Lohse et al., 2011; Lohse and Sherwood, 2012; Greig and Marchant, 2014; Marchant and Greig, 2017). An external focus has also shown superior performance over an internal focus for peak torque (Greig and Marchant, 2014), force production (Marchant et al., 2009; Halperin et al., 2016), reduced pre-movement time in early stages of learning an isometric force production task (Lohse, 2012), accuracy in a force production task (Lohse et al., 2011; Lohse and Sherwood, 2012), more repetitions before failure (Marchant et al., 2011), and better movement kinematics for the snatch (Schutts et al., 2017).

**Table 4:** Characteristics of the aims, conditions, measures, and key findings.

**References**	**Aims**	**Conditions**	**Measures**	**Key findings**
Calatayud et al., 2018a	To investigate the effect of different attentional focus strategies on muscle activity during the bench press at explosive and controlled speeds.	Focus instruction: regular focus (lift the barbell in a regular way), focus on pectoralis (try to focus on using your chest muscles only), focus on triceps (try to focus on using your triceps muscles only) Barbell speed: controlled (2 s rate of descent 2 s ascent), explosive (as fast as possible)	Electromyography (EMG) Contraction duration (for explosive condition)	During the controlled condition, focusing on either the pectoralis or triceps resulted in increased EMG activity in the pectoralis by 6 and 4%, respectively, over using a regular focus. Additionally, in the controlled condition, activity in the triceps increased 4% when a triceps focus was used. There was no difference found in EMG activity or contraction duration between the focus conditions when lifting explosively.
Calatayud et al., 2018b	To investigate the effects of either external or internal focus strategies and varying grip widths on muscle activity during the bench press	Focus instruction: internal for pectoralis (try to focus only on using your chest muscles), internal for triceps (try to focus only on using your triceps muscles), external focus (just lift the barbell in a regular way)	EMG	Significant main effects for attentional focus and grip width for EMG activity in both the pectoralis and triceps muscles (higher during internal focus than external focus), but no significant interactions.
Greig and Marchant, 2014	To investigate the effects of internal and external focusing instructions on force production and muscle activity at varying movement speeds.	Focus instructions: internal (focus on the movement of your arm and muscles during the lift), external (focus upon the movement of the crank hand-bar during the lift). Speed: move the crank at 60°, 180°, or 300° per second.	Force production measured using peak torque Muscle activity measured using EMG	External focus associated with lower EMG in all speeds compared to internal focus. However, an external focus only produced greater torque than in an internal focus when speed was at 60° per second condition. This suggest that focusing instructions may be less effective for explosive movements.
Halperin et al., 2016	To investigate the effect of attentional focus on a force production during an isometric midthigh pull in trained athletes.	Focus instructions: control (Focus on going as hard and as fast as you possibly can), internal (Focus on contracting your leg muscles as hard and as fast as you possibly can), external (Focus on pushing the ground as hard and as fast as you possibly can)	Force measured using Newtons (N).	Both the external focus and control instructions resulted in greater force production than the internal focus instructions (9 and 5%, respectively). This suggests that adopting an internal focus while exerting maximal force hinders performance.
Kristiansen et al., 2018	To compare the effect of internal and external focus with no focus instruction on muscle activity during a 60% 3 RM bench press	Three conditions completed by all participants. Baseline included no focus instructions. External condition instructed participants to focus on the movement of the barbell and that the movement should be as smooth as possible. Internal condition required participants to focus on the contraction of the pectoralis muscle.	Electromyography (EMG)	Both internal and external focus conditions resulted in significantly greater mean and peak EMG amplitudes for 6-upper body muscles compared to baseline, despite all conditions involving the same weight and repetitions. These results suggest that both focus instructions were detrimental to performance. Results could be explained by the fact that participants were experienced lifters and introducing complicated instructions may have interfered with their natural technique.
Lohse, 2012	To investigate the effect of attentional focus on accuracy and pre-movement time during an isometric force production task	Focus instructions: External focus (Mentally focus on the push of your foot against the platform and push harder or less on the platform), Internal focus (Mentally focus on the calf and contract the muscle harder or less)	Accuracy (needed to meet maximum voluntary force [MVC] of either 25% or 50%)	External focus resulted in reduced pre-movement time in early stages of learning, and improved transfer performance (moving from 25% MVC target to 50% MVC target or vice versa) over internal focus.
Lohse et al., 2011	To investigate the effect of attentional focus instructions on force accuracy and muscle activity during an isometric force production task	Focus instructions: External focus (Mentally focus on the push of your foot against the platform), Internal focus (Mentally focus on pushing with the muscle of your calf)	Accuracy (target = 30% of maximal force) EMG	Greater accuracy in the external focus condition, and less muscle activity in the tibialis anterior of the calf, but no difference in activity in the soleus. These results suggest that muscles were more efficient and performed better when using an external focus than using an internal focus.
Lohse and Sherwood, 2012				
Experiment 1	To test the effects of attentional focus on accuracy and efficiency at varying levels of muscle contraction	Focus instructions: External focus (mentally focus on the push of your foot against the platform), Internal focus (mentally focus on pushing with the muscle of your calf) Target force: 30, 60, and 100 %MVC. Participants were informed how this force would translate to pounds of force. Force held for 4 second windows.	Accuracy (absolute error: average force across 3 s window with participant's target force subtracted) Cocontraction-ratio (dividing tibialis activity by soleus activity)	External focus produced more accurate force production across all force production targets. Additionally, an external focus reduced cocontraction, suggesting that the muscles performed more efficiently.
Experiment 2	To test the effects of attentional focus on muscle fatigue at varying level of muscle contraction	Focus instructions: External focus (mentally focus on the push of your foot against the platform), internal focus (mentally focus on pushing with the muscle of your calf) Target force: 30, 60, and 100 %MVC. Participants were informed how this force would translate to pounds of force. Participants were required to hold the target force for 60s for 30 and 60 %MVC or until failure for 100 %MVC.	Accuracy (absolute error: average force across 3 s window with participant's target force subtracted) Cocontraction-ratio (dividing tibialis activity by soleus activity) Time to failure (length of holding 100% MVC) Ratings of perceived exertion (for 100% MVC)	Attentional focus had no effect on time to failure, RPE, or accuracy. However, an internal focus of attention resulted in greater cocontraction in early trials, suggesting less efficient muscular coordination.
Marchant et al., 2008	To investigate the effect of attentional focusing on muscular activity during the bicep curl with controlled movement speed	Focus instructions: no instruction, internal focus (focus upon the movement of the arm during the lift), external focus (focus upon the movement of the crank handle during the lift)	Peak EMG Activity Integrated/total EMG activity over 10 repetitions	Peak muscle activity was lower when using external focus than using internal focus and no specific instructions. Total muscle activity was also lower in the external condition than internal condition. These results suggest that the use of external focus resulted in more efficient muscle control.
Marchant et al., 2009	To investigate the influence of attentional focusing instructions on force production and muscle activity during isokinetic elbow flexions.	Focus instructions: internal (focusing internally onto movement mechanics), external (focusing externally onto the outcome of the movement)	EMG Force production (torque)	An external focus of attention results in greater force production and lower EMG activity than an internal focus.
Marchant et al., 2011	To investigate the influence of attentional focusing instructions on muscular endurance in three types of exercises in experienced athletes	Focus instructions: Control (perform as many repetitions as you can before failure), internal (focus on moving and exerting force with your arms/legs), external (focus on moving and exerting force through the barbell)	Repetitions until failure	For the assisted bench press, an external focus of attention resulted in more repetitions before failure than an internal focus, but not for the control instructions. For the standard bench press and back squat, using an external focus of attention resulted in more repetitions before failure than both the internal focus and control instructions.
Marchant and Greig, 2017	To investigate the effect of internal focus instructions which emphasize specific muscular activity compared to external focus instructions which emphasize outcome on force and muscle activity during a knee extension task.	Focus instructions: internal focus (focus on muscular activation), external (focus onto the movement outcome)	Force (Peak torque and mean power output) Integrated EMG of vastus lateralis (VL), vastus medialis oblique (VMO), and rectus femoris Ratio of activation for VMO and VL	No difference in torque produced between the focus instructions. External focus instructions resulted in lower iEMG magnitude across muscles than internal focus. Internal focus resulted in greater EMG activity, but not in the specific VMO, suggesting that the internal focus did not result in a selective isolation. Instead, there was a spreading activation effect with elevated activity in muscles not within the focus of attention. Findings suggest that an external focus of attention results in increased muscular efficiency.
Neumann and Heng, 2011	To investigate the effects of an associative and dissociative attentional strategy on muscle activity for a biceps curl	Focus instructions: control (repeat previous lifting technique), dissociative (listen to lyrics of a song playing, and count the occurrence of a word), associative (attend to an auditory tone which varied based on EMG from biceps activity)	EMG and iEMG Heart rate Perceived exertion and exercise satisfaction Movement degrees and velocity	Adopting an associative strategy resulted in lower EMG, iEMG, and heart rate compared to dissociative and control strategies. No difference found in subjective measures of exertion or satisfaction. Movement velocity slower in associative condition than in dissociative or control condition.
Schutts et al., 2017	To investigate the effect of focus of attention on kinematic performance of the snatch	Focus instructions: internal focus (concentrate on moving your elbows high and to the side rapidly), external focus (concentrate on moving the barbell back and up rapidly)	Barbell-cervical-hip angle Vertical/Horizontal barbell velocity Peak elbow velocity	Internal focus resulted in increased elbow velocity compared to external focus. External focus increased horizontal barbell velocity compared to internal. The internal focus also resulted in the athlete squatting under the barbell too soon.
Snyder and Fry, 2012	To investigate the ability of athletes to isolate specific muscles when given internal focus instructions during the bench press	Focus instructions: non-specific instructions, internal (focus on chest muscles), internal (focus on arm muscles)	EMG	Instruction to focus on the chest muscles and triceps muscles increased muscle activity over baseline in these specific areas when bench pressing 50% of 1-RM. At 80% of 1-RM only instructions to focus on chest muscles resulted in an increase in muscle activity over baseline, while instructions regarding a focus on triceps resulted in no further activity over baseline.
Vance et al., 2004				
Experiment 1	To investigate the effect of focus of attention on movement speed and muscle activity during the biceps curl	Focus instructions: internal (concentrate on biceps muscles), external (concentrate on the curl bar)	Angular velocity, EMG and iEMG	Movement was faster and iEMG was reduced in the external focus condition compared to the internal focus condition.
Experiment 2	To investigate the effect of focus of attention on muscle activity when timing is controlled during the biceps curl	Focus instructions: internal (concentrate on biceps muscles), external (concentrate on the curl bar)	Angular velocity, EMG and iEMG	iEMG was reduced when adopting an external focus compared to an internal focus. This was true even with average range and movement time the same between focus conditions.

The conditions that may limit the effects of an internal or external focus have also been examined. Lifting at a controlled or explosive speed did not alter the size of muscle contractions as measured by EMG for an internal focus strategy (Calatayud et al., 2018a,b). Moreover, using grips of different width does not interact with the type of attentional foci (internal or external) on EMG activity. In a study on force production, an external focus of attention produced lower EMG than an internal focus at all speeds, but an interaction between focus type and speed was observed for peak torque such that the attentional focus conditions differed in torque only at slower speeds (Greig and Marchant, 2014). The latter findings suggest that lifting speed may influence attentional focus effects.

It is often reported that an external focus is superior than both an internal focus and a control (no instructions) condition and that this is evidence for a beneficial effect of an external focus rather than a relative detrimental effect of an internal focus (Wulf, 2007). Similar outcomes have been reported in weightlifting and force production tasks (Marchant et al., 2008, 2011). However, this finding has not always been found. Both an external and control condition resulted in greater force during an isometric midthigh pull than an internal condition (Halperin et al., 2016). An external focus resulted in more repetitions to failure than an internal focus, but did not differ from a control condition for an assisted bench press (Marchant et al., 2011).

Furthermore, research has not always shown performance benefits with an external focus of attention. No differences between internal and external foci have been observed for time to failure or ratings of perceived exertion for a long duration force production task (Lohse and Sherwood, 2012). In different findings, Kristiansen et al. (2018) reported that both an external and an internal attentional focus produced greater mean and peak EMG amplitude than a baseline condition during a bench press. In the baseline condition, participants performed the lift as they normally would. The authors suggested that the results may reflect that experienced weight lifters were participants and that the use of attentional instructions of any type may have interfered with their normal technique. Another explanation could be that the baseline condition was completed first and performance in the subsequent conditions suffered from fatigue effects. Yet another explanation may relate to the relative high complexity of the attentional focus instructions. For instance, the external focus instructions required participants to maintain the same tempo of the lift as done in the baseline condition while also attending to the movement of the barbell and making the move as smooth as possible. The internal focus instructions also referred to moving the barbell as smooth as possible and at the same tempo as the baseline condition, as well as focusing on the pectoralis muscle contractions. These internal focus instructions included some reference to an external focus (move the barbell as smooth as possible). Wulf (2007) has suggested that the use of vague or complex attentional focus instructions may mitigate the benefits of an external focus over an internal one.

To elicit an internal attentional focus, researchers have typically used simple instructions requiring participants to attend to the feelings of the muscle or combinations of muscles primarily involved in the lift. A focus on the primary muscles involved in lifting will increase EMG activity measured from that muscle (Calatayud et al., 2018a). Moreover, a focus on secondary muscles for a lift (e.g., triceps for a bench press) will increase EMG activity of the primary muscle (i.e., pectoralis). However, it should be noted that effects of focusing on a specific muscle may vary across the weights being lifted. Attention to a specific muscle increased activity of the muscle that attention was directed toward when a lighter weight was lifted (50% of 1-RM) but not when a heavier weight was used (80% of 1-RM) for a bench press (Snyder and Fry, 2012).

Instructions used to induce an external focus of instruction have typically required participants to focus on the movements of the barbell, dumbbell, crank handle, or platform (see Table 4). Calatayud et al. (2018b) defined an external focus as lifting the barbell in a regular way. However, it may be argued that this instruction did not adequately require participants to focus on the movement effects of the exercise. Further research would be required to evaluate this possibility.

In the only research to examine other forms of attention focus strategies, Neumann and Heng (2011) compared an associative and dissociative focus strategy during a biceps curl task. The study was also unique in measuring heart rate in addition to muscle activity (see Neumann and Thomas, 2009, 2011 for examples of attentional focus effects on heart rate during sport tasks). The dissociative condition required participants to listen to audio of a song whereas the associative condition consisted of listening to audio of a tone that changed in nature based on the EMG amplitude recorded from the biceps muscle. A control condition using no audio and no specific focus instructions was also used. The results showed that EMG, iEMG, and heart rate were lower during the associative strategy than during the dissociative strategy and control conditions. The differences between conditions may reflect a relative benefit of an associative strategy for muscular efficiency. The benefit may reflect that the associative condition had a predominantly external focus (i.e., the effects of the movement on the external audio stimulus). However, the associative condition may have also had an internal component due to the audio being directly linked to muscle contraction strength.

### Theoretical Frameworks

The beneficial effect on performance of adopting an external focus of attention compared to an internal focus is well-established across a range of different motor tasks, including those that are sport-related (Wulf, 2013; Wulf and Lewthwaite, 2016). The same conclusion has been reached in most, but not all, of studes examining internal and external attentional foci during weightlifting tasks (see Table 4). In addition, it has been shown that adopting an associative strategy results in beneficial effects over a dissociative strategy for bicep curls (Neumann and Heng, 2011), which may reflect that the associative condition in the study was largely external in nature.

The benefits of an external focus over an internal focus of attention in terms of reduced muscle activity may be explained by differences in the spread of activation between the two types of foci. In a knee extension task, Marchant and Greig (2017) reported that an internal focus of attention produced higher overall EMG, and that this was not specific to the muscles isolated in the task but that it reflected a spreading activation of increased muscle activity. The authors suggested that this pattern reflects than an external focus of attention results in increased muscular efficiency. A similar interpretation has been made using the measure of integrated EMG (iEMG) (Vance et al., 2004). An external attentional focus has resulted in lower iEMG than an internal focus (Vance et al., 2004; Marchant and Greig, 2017).

Increased muscular efficiency is a key component of the constrained action hypothesis (Wulf et al., 2001), which is one framework in which prior research has been based on. The constrained action hypothesis proposed by Wulf et al. (2001) suggests that adopting an external attentional focus promotes automatic, natural movement control, whereas adopting an internal attentional focus disrupts this automaticity and constrains the neuromuscular system (Wulf, 2013). When an individual focuses on an external cue, it facilitates attention to stimuli distant from their body. This then allows automatic behavior to dominate, improving performance. In contrast, an internal cue constrains motor control, reducing performance. The constrained action hypothesis has since been supported by a number of studies in several different contexts.

Initial evidence for the constrained action hypothesis came from a dynamic balancing task using a stabilometer (Wulf et al., 2001). Participants given an internal instruction were told to focus on their feet and to keep them horizontal, while participants given an external instruction were told to focus on markers attached to the balance platform. Participants underwent 2 days of practice, with each day consisting of seven 90 s balance trials, followed by a retention test on the third day in which no focus instructions were given. Performance was measured on three measures, including reaction time to a dual-task procedure, balance performance, and frequency of adjustments. Participants given the external cue had significantly quicker reaction times, better balance performance, and higher frequency of adjustments. This suggests that those given the external cue experienced lower attentional demands, better learning of balance, and less disruption from voluntary attempts to correct posture, respectively.

McNevin et al. (2003) expanded on these findings in a similar balancing task by introducing different levels of external focus by varying the distance from the body on which participants were instructed to focus. Four groups of participants were instructed to focus on their feet (internal), markers close to the feet (near), markers in the center of the balance platform (far inside), and markers on the outside of the platform (far outside). Findings were similar to that of Wulf et al. (2001) with all three external foci groups performing better than the internal focus group. Additionally, the far inside and far outside groups showed a higher frequency of adjustments, demonstrating the use of more natural automatic motor control.

Most recently, Vidal et al. (2018) investigated the constrained action hypothesis using a standing long jump task with internal and external focus instructions. As expect, participants given an external focus of attention jumped significantly further than those given an internal focus instruction. Additionally, attention instructions affected the movement strategy used by participants, with a difference found in ankle-knee coordination. Those given an internal focus to extend their knees as rapidly as possible showed a jump that recruited primarily knee movement, with minimal hip or ankle movement. In contrast, those given an external focus of trying to jump to cones placed in the distance showed good ankle-knee coordination. These results suggest that the internal focus constrained participants to employing knee flexion, whereas an external focus allowed an automatic coordinated movement pattern between knee and ankle.

The constrained action hypothesis has provided a good explanation of findings from motor tasks as well as weightlifting tasks. Attempts have been made to integrate the hypothesis with other notions of attentional focus effects at the neuromuscular level (see Lohse, 2012; Lohse and Sherwood, 2012). For example, (Willingham, 1999) Control Based Learning Theory of motor control (COBALT) suggests that there are stages of processing that can operate through explicit or implicit modes of control when performing a motor task. Implicit modes are advantageous because they promote automatic selection of spatial targets and automatic movement sequences. An external focus of attention may thus promote implicit control of motor actions and result in better performance. The nodal-point hypothesis (Hossner and Ehrlenspiel, 2010) is another notion with similarities to the constrained action hypothesis. This hypothesis suggests that attention serves to select appropriate actions through the selection of sensory feedback and making ongoing corrections to movements in response to this feedback. This process is faciliated when attention is directed to the effects of movements rather than the movement execution itself. Further research is required to examine the links between the various theories of motor performance within a weightlifting context.

## Discussion

The research conducted to date has potentially important implications for training and performance of weightlifting tasks. The increased muscular efficiency and accuracy of force production with an external focus of attention over no specific focus or an internal focus suggests that athletes should adopt an external focus during competition. An external focus may result in superior performance to allow the athlete to lift a heavier weight than may otherwise be possible if attention is directed in other ways. Athletes should practice adopting an external focus when simulating competition during training so that it becomes a component of their competition lifting routine.

Conversely, if increased activation of muscles is the desired goal, there is an argument that athletes should adopt an internal focus of attention. Such benefits of an internal attentional focus would typically exist for training programs that aim to increase muscle growth or strength gains (Marchant et al., 2008). The increased activation of the muscle is likely to be observed in the muscle attended to and to spread to other muscles involved in the lift as suggested by the findings of Marchant and Greig (2017). A similar effect of increased muscle activation might also be observed if athletes adopt a dissociative attentional focus, based on the findings of Neumann and Heng (2011). Whether adopting an internal or dissociative strategy has any actual beneficial effect in training in the short or long term (e.g., increase muscle fatigue more quickly or lead to increased gains in strength) remains to be determined.

An important practical consideration is to determine exactly how the benefits of an external focus of attention for sport performance (or an internal focus on muscle activation) can be achieved in practical terms. The first step in developing effective strategies is to identify what are the key elements that athletes should direct their attention to (Marchant et al., 2008). In research conducted to date, an external focus has been effective when attention is directed to bar or weight being lifted. However, some weight training exercises does not use any apparatus (e.g., unweighted squats, sit ups). In cases when there is no specific implement or object used in a sport, Wulf (2007) suggests analogies and metaphor could be used. Neumann and Brown (2013) had participants direct their attention externally during a sit up task by asking them to focus on making smooth movements without any reference to a body part. For an internal focus of attention, the muscle (e.g., bicep, pectoralis) or the body part (foot, legs) has been commonly used in research as the focal point of attention.

The second consideration is the mechanism by which an instructor promotes an increased attentional focus. The use of instructions, as done in research to date, is the simplest approach and has shown to be effective. Coaches can work with athletes by using instructions to provide clear guidance on how to direct attention effectively. An external focus can be promoted by directing athletes to focus on visual cues like bar movement, the sound of the machine, pushing against the bar, or the end result of the lift. An internal focus can be promoted by instructions that direct athletes to focus on muscle tension, body movements, technique, and form.

Coaches should also provide feedback to athletes to reinforce their learning. Feedback might be enhanced by using additional cues to provide information. For an external focus of attention, this might involve placing markers on the bars or weights, using mirrors, making video recordings, or attaching sensors to the bar or weights to measure movement dynamics (acceleration, velocity, smoothness). For an internal focus, EMG recordings of muscles or movement sensors attached to the body can be used provide visual or auditory feedback to athletes. In addition, athletes should be aware that their own use of “psyching up” or self-talk might need to be modified to ensure that the appropriate attentional focus is used. For example, cue words like “strong” and “powerful” might inadvertently direct the athlete to focus their attention internally and should be modified accordingly during competition.

The third consideration is how to tailor the approach to the specific context. In simple lifts, like the biceps curl, attentional focus instructions are relatively simple because the movement is constrained. However, compound lifts will involve multiple muscles and limbs. In addition, multiple component lifts like the clean and jerk involve discrete movements performed in sequence. Internal focus instructions might need to be varied according to the stage of the lift. Whether external focus instructions need to be varied across the lift remains to be determined. Based on the notion that external attentional focus benefits might result from both intramuscular efficiency and intermuscular efficiency (Vance et al., 2004) the adoption of a single focus may be the most beneficial throughout. For the clean and jerk, for example, the athlete would merely focus on exerting force on the barbell at all stages of the lift. Importantly, when research has examined different types of lifts, the results suggest that the benefits of an external focus of attention may become more pronounced as the movement complexity increases (Marchant et al., 2011). Another important consideration is the amount of weight being lift. Attentional focus instructions may be less effective with higher intensity lifts (Snyder and Fry, 2012). The use of very simple instructions, or just cue words, and extensive practice at lighter weights might mitigate the negative impact of heavy weights on attentional focus effects. Indeed, Schutts et al. (2017) recommends that in general coaching cues for lifting are best if they are short, concise, and specific to the key element being trained.

Exercise psychologists and fitness trainers might also consider appropriate psychological strategies in attentional focus for recreational exercisers. For instance, an external focus of attention may benefit recreational exercisers to adhere to exercise programs. This is because an external focus could draw attention away from negative cues associated with physical exertion and toward positively reinforcing outcomes of the weightlifting exercise (e.g., completion of a rep or set). Research in our laboratory has shown that an external attentional focus has benefits to physical and psychological states during cardiovascular exercise tasks (e.g., Neumann and Piercy, 2013). Moreover, external attentional focus strategies may complement dissociative focus strategies like listening to music, in promoting exercise adherence. An external associative focus may be particularly beneficial given that dissociation can be more difficult to maintain at high levels of exercise intensity. Cognitive strategies like attentional focus may also be integrated with other approaches to promoting physical exercise, like goal setting (e.g., Salehian et al., 2011; Neumann and Honke, 2018).

The studies examined in the present review used weightlifting tasks that were typically of short duration by limiting the number of repetition and sets performed. In considering their findings of less efficient muscular coordination with an internal focus than an external focus, Lohse and Sherwood (2012) suggested that their findings could be relevant to endurance type tasks such as long distance running. It was noted that even a small difference in stride efficiency could be magnified over the course of the entire race due to the high repetitions of the movement. They noted that their internal focus condition produced a trend for shorter time to failure than the external focus condition and that this could reduce performance in endurance running. The benefits of an external attentional focus over an internal attentional focus have been demonstrated in running tasks of short duration (e.g., Schücker et al., 2009; Neumann and Piercy, 2013). However, in a time to exhaustion running task, no difference between internal and external foci was observed in performance or physiological variables (Vitali et al., 2019). Further research is thus required to examine the effects of different attentional foci at the neuromuscular level for endurance tasks and for weight lifting tasks that are performed to exhaustion.

Further research can be conducted to extend upon research regarding the potential benefits of adopting an external (or internal) attentional focus. Importantly, the studies conducted to date have typically been conducted in single-session designs. Thus, the long-term benefits of an external attentional focus remain to be determined. Similarly, transfer effects need to be established to determine whether beneficial attentional focus instructions practiced with one type of lifting exercise in the laboratory will transfer to real world training or competition or to other types of lifting exercises. In addition, it would be worthwhile to examine whether transfer occurs to similar sporting tasks. For example, some sports like shot put and discus, require a short-term maximal muscular effort. It would be informative to examine whether training in an external focus of attention transfers to these tasks (Vance et al., 2004).

The use of new technology to induce attentional foci or that provide additional contexts in which weightlifting can occur requires further investigation. Virtual reality (VR) has emerged as a technology applied to sport (for reviews see Neumann, 2016, 2019; Neumann et al., 2018) and has been most commonly applied to cardiovascular exercises (Murray et al., 2016; Neumann and Moffitt, 2018; Parton and Neumann, 2019) but has also been examined with weightlifting. Chen et al. (2015) examined weightlifting in a virtual environment and found that bicep muscle activity and ratings of perceived workload during bicep curls was higher in the VR condition than a non-VR. These findings may reflect that the VR condition increased an internal attention focus on the mechanics of the movement within the virtual environment. The substitution of a weighted apparatus that participants must move to produce an effect in a virtual environment would be one way to promote an external attentional focus (e.g., completing a deadlift could be translated into virtually lifting a heavy bar to free a trapped virtual person). Physiological measures can also be applied in sport in the form of biofeedback. The study by Neumann and Heng (2011) is an example where muscle activity was translated into an audio signal to direct attentional focus during a biceps curl. In addition, physiological measures other than EMG could be used to examine attentional focus effects on general arousal or emotional reactivity due to the relationship between central and peripheral measures with emotional states (e.g., see Neumann and Westbury, 2011).

## Conclusions

Weightlifting for physical conditioning or sport depends on many physical and psychological factors. Research examining cognitive strategies has shown that performance at the neuromuscular and behavioral level are influenced by the attentional foci that an athlete adopts. To maximize muscular efficiency, an external focus of attention is more optimal than an internal focus of attention or no specific focus in most cases. The challenge remains for researchers to further explore this effect and determine under which conditions it may be magnified. This information will assist in translational research that can allow athletes to reach a higher level of performance than might otherwise be possible.

## Author Contributions

The author confirms being the sole contributor of this work and has approved it for publication.

### Conflict of Interest Statement

The author declares that the research was conducted in the absence of any commercial or financial relationships that could be construed as a potential conflict of interest.
